# Wood ants as biological control of the forest pest beetles *Ips* spp.

**DOI:** 10.1038/s41598-021-96990-5

**Published:** 2021-09-09

**Authors:** Gema Trigos-Peral, Orsolya Juhász, Péter János Kiss, Gábor Módra, Anna Tenyér, István Maák

**Affiliations:** 1 Museum and Institute of Zoology, Polish Academy of Sciences, Wilcza Street 64, 00-679 Warsaw, Poland; 2 Department of Ecology, University of Szeged, Közép fasor 52, Szeged, 6726 Hungary; 3 Doctoral School in Biology, Faculty of Science and Informatics, University of Szeged, Közép fasor 52, Szeged, 6726 Hungary; 4 Doctoral School of Environmental Sciences, University of Szeged, Rerrich Béla Square 1, Szeged, 6720 Hungary; 5 Department of Physical Geography and Geoinformatics, University of Szeged, Egyetem Street 2-6, Szeged, 6722 Hungary

**Keywords:** Conservation biology, Ecological networks, Ecosystem ecology, Forest ecology, Forestry

## Abstract

Climate change is one of the major threats to biodiversity, but its impact varies among the species. Bark beetles (*Ips* spp.), as well as other wood-boring pests of European forests, show escalating numbers in response to the changes driven by climate change and seriously affect the survival of the forests through the massive killing of trees. Many methods were developed to control these wood-boring beetles, however, their implementation can be detrimental for other forest specialists. Ants are widely used for biological pest-control, so in our study, we aimed to test the effect of *Formica polyctena* on the control of the wood-boring beetles. The results show that the proportion of infested trees is significantly reduced by the increase of the number of *F. polyctena* nests, with a strong effect on those infested by *Ips* species. We also show that the boring beetle community is shaped by different biotic and abiotic factors, including the presence of *F. polyctena* nests. However, the boring beetle infestation was not related to the latitude, altitude and age of the forests. Based on our results, we assert the effectiveness of the red wood ants as biological pest control and the importance of their conservation to keep the health of the forests.

## Introduction

Beetles are the most diverse group of insects on Earth with about 400,000 described species^1^. This large group is distributed worldwide and their species are present in all the ecosystems^2,3^, being involved in many different types of interactions. Some species belonging to the group of wood-boring beetles destroy the wood either to obtain food (xylophagous) or to protect their eggs, larvae, and pupae^4^. Wood-boring beetles play an important role in forests due to their contribution to dead wood degradation, involvement in the nutrient cycle, and facilitation of the arrival and establishment of other rare insect species^5–7^. However, some of the xylophagous beetle species are known to cause serious damages in fruit plantations and forests^8–10^.

Wood-boring beetle species belong mainly to the families *Cerambycidae, Buprestidae*, and *Curculionidae*, which altogether encompass a vast list of the species considered main forest pests^11^. Wood-boring beetles complete their life cycle by establishing a parasitic interaction with the tree^11,12^. Some of the longhorn beetles (*Cerambycidae*), jewel beetles (*Buprestidae*), and bark beetles (*Curculionidae, Scolitynae*) are monophagous or oligophagous, meaning that they are dependent on one or a few tree species of the same genera^13^. Wood-boring beetles are very important components of the nutrient cycle in forests as they decompose the decaying wood, contribute to the regeneration of soil nutrients and facilitate the establishment of other insects^14^. Usually, they infest living but weakened trees or untreated lumber, in which females (attracted by volatiles produced by dying or recently dead trees) place their eggs in crevices of trees affected by water stress or roots damaged by biotic or abiotic factors^8,11^. Moreover, they can also kill conifer seedlings by feeding on their stem bark^15–17^. In temperate coniferous forests, since infestations can potentially kill the trees, these wood-boring species constitute serious pests^5^ and can cause enormous ecological^18,19^ and economic losses^8,20^.

The most harmful wood-boring beetle species throughout the world belong to the subfamily *Scolytinae* (Latreille, 1804), which are considered the most destructive forest pests^21–23^. In Europe, the bark beetle *Ips typographus*, known as  the European spruce bark beetle, is the cause of many major economic losses in the forest industry^12,24^ and forest degradation^25–27^. The massive loss of trees is caused by the bark beetle tunnelling under the trees’ bark and chewing on the cambium layer in the middle and lower parts of the trees that reduce the capacity of the tree to produce new cells that allow the growth of the trunk, branches and roots; but also by the indirect negative effect of fungi, bacteria, and mites associated with bark beetles^28^. Moreover, in European spruce forests, large-scale windthrow is usually connected with outbreaks of *I. typophagus* and results in extensive loss of trees^7^. Such outbreaks of the pest species are caused mostly by changes in the natural or semi-natural environment that can be linked to anthropogenic activities, having a strong impact also on the insect communities^29–32^.

Human activities lead to changes in many environmental factors, such as the increase of the global temperature linked to the climate change that threatens the survival of many insect species^18,33^. The short generation time and the close relation between temperatures and life-history traits^18^ make insects especially sensitive to temperature changes, like is the case of the specialist ants in the *Formica rufa* group, oligotope species of coniferous and mixed forests^34^. Besides the changes in temperatures, human impact also induces habitat loss and other changes in the natural environment, such as alterations in the precipitation regimes that affect the forest growth and production^35–37^. However, climate change can have an opposite effect in some invasive and pest species^38^, like the bark beetles, and influence positively their populations as it can shorten their generation time and facilitate their spread that leads to the outbreak of their native populations^25,27^. To try to overcome the negative effects, different management techniques have been developed in order to facilitate an early detection to minimalize the damage caused by different pests^24,25^. However, these techniques do not always take into consideration the long term effects and the well-being of many other forest-dwelling species, like birds (as woodpeckers^39^), parasitoids^40^ or other arthropods (as the clerid beetle *Thanasimus dubius*^41^) including wood ants^37^.

The natural ecosystems tend to find the balance among all the levels by keeping an equilibrium between preys and predators^42^, a concept that has become the basis of biological pest control. Under such circumstances, the biological control of wood-boring pest species can be more efficient with the use of native enemies together with the current management practices. Ants are known as natural enemies of insect pests worldwide^43,44^. In European mixed forests, red wood ants shape the invertebrate communities through different interactions at multi-trophic levels^45,46^ and have been also used as a biological control for pests in plantations since they are considered to be able to prey on or deter arthropod pests^14^. Moreover, they feature a series of characteristics that make them very efficient in this matter, such as their vast abundance in many ecosystems, a quick reaction to the increasing prey abundance, and the effective retrieval of prey individuals. In the case of red wood ants, the large size of their nests and their organization in polydomous systems allows them to host an enormous number of individuals^34^ and broods in the inhabited area, increasing considerably the protein consumption of the population. Considering the former, in our study, we investigated whether the presence of the red wood ant *Formica polyctena* contributes to the forest ecosystem balance by indirectly reducing the death of trees through decreasing the parasite infestation of trees. We hypothesized that the presence of *F. polyctena* in forests will influence the proportion of infested trees by bark beetles and other wood-boring beetle species, but also the fungal infestation linked to these beetles. To test the role of this wood ant species as a biological control agent, we carried out a large-scale study in which we measured the size and number of *F. polyctena* nests, the number of infested trees and the cause of their infestation (type of wood-boring beetles or fungus), as well as the physical damages of the trees, and we expected a reduced negative impact of wood-boring beetles in forests with larger populations of wood ants. Additionally, we made an attempt to contribute to the knowledge about the relevant ecological role of red wood ants in forest ecosystems and highlight the importance of the *Formica rufa* group in habitat conservation and forest pest management.

## Materials and methods

The sampling was performed during the summer months (June-July) of 2017 and 2019, and was carried out in 31 forest patches from 12 regions located across a latitudinal gradient (46.215283° N–54.069650° N) crossing three different Central-European countries (Hungary, Slovakia, Poland). The lowest latitude corresponded to Hungary, where two regions (Ásotthalom and Kiskunság) were sampled. At the middle latitude of our gradient, we sampled the Carpathians, with Mátra and Bükk Mountains in the Hungarian area, Fatra Mountains in the Slovakian area, and Tatra, Pieniny, and Gorce Mountains in the Polish area. The other sampled areas encompassed the middle of Poland to the Baltic Sea and corresponded to Świętokrzyska, Kampinos, Białowieza, and Koszalin at the highest latitude.

In each region, we selected mixed forests that are under forestry management. These study sites are composed of *Picea abies*, *Pinus sylvestris*, *P. nigra*, *Abies alba* and *Larix decidua* as the most common coniferous species, and *Betula pendula*, *Robinia pseudoacacia* and *Quercus* spp. as the most common non-coniferous ones (according to the local forestry information: NÉBIH, Mapový portal KIMS, Bank Danych o Lasach).

To carry out the sampling, we used the colonies located haphazardly after intensive searching in the study regions. The location of the central point was marked with GPS (GARMIN Oregon 700t), and the 150 × 150 m plots around them were assigned in a random manner using Google Earth Pro. This plot size was determined to be a good representation of the territory of *F. polyctena*^47^. In accordance with the presence of *F. polyctena* populations, in most regions (Ásotthalom, Kiskunság, Bükk, Fatra, Pieniny, Świętokrzyska, Kampinos, Białowieza), we could sample three plots, while in Gorce, Tatra and Mátra Mountains we found only two plots, and in Koszalin only one plot. Within the plots, we recorded the GPS location of each *F. polyctena* nest and measured their dimensions (two perpendicular diameters and the height). The above-ground nest volume (i.e., semi-ellipsoid) was determined using the following equation:

$$V = \frac{{0.75*\uppi *{\text{r}}_{1} *{\text{r}}_{2} *{\text{h}}}}{2}$$

where h is the height of the nest, and r_1_ and r_2_ are the two perpendicular nest radii. We used this equation because the above-ground nest volume is closely related to the red wood ant colony size^48–50^.

To test the relationship between the presence of *F. polyctena* nests and the tree infestation, we recorded the number and GPS location of every infested tree within a plot and noted down the type of parasite infestation (bark beetle, longhorn beetle, jewel beetle, or fungus). For a more precise characterization of the health status of forests, we also recorded the status of the infested trees (Alive or Dead) and their position (Standing or Laying). Finally, we determined the latitude and altitude of the sampling sites with the help of Google Earth (Google 2019) and established a “latitude value” as an increasing distance value starting from the southernmost study region (Ásotthalom: value 1) and added + 1 value with every 20 km (in beeline) passing to the north, finishing in Koszalin (North Poland, value 61; 28). In a previous study, we found a negative correlation between the latitude and the large-scale environmental variables like irradiation and temperature^48^. The average age of the forests was gathered from local forestry databases (NÉBIH, Mapový portal KIMS, Bank Danych o Lasach).

### Statistical analysis

First, we were interested in whether the degree of the beetle infestation is correlated with the latitudinal and altitudinal gradient of the study sites and the forest age. To test this, we built separate GLM models (negative binomial error, maximum likelihood fit) for each parasite group with the proportion of trees infested by *Ips* spp., Cerambycidae Buprestidae and fungi as dependent variables, whereas the log + 1 transformed latitude value, altitude, and age of the forest as explanatory variables (N = 12).We used a Redundancy Analysis with Hellinger transformation of species data (RDA, N = 31) to test the association between the number of trees affected by both biotic and abiotic factors. In the model, a matrix built with the average frequency of the trees affected by each beetle and fungi species from each study plot within the study sites was used as a dependent variable. The matrix was built with log-transformed averages of the following explanatory variables: number of nests (Nests number), nests size (Nest volumes), the perimeter of the tree trunks (Perimeter), the ratio of alive trees to the total number of affected trees (Alive), the ratio of the standing trees to the total number of affected trees (Stand), and the ratio of conifers to the total number of affected trees (Conifer). The significance tests for the general model, in order to calculate the effect of the explanatory variables on the dissimilarity matrix of the dependent variables (number of  *Ips spp.*, Buprestidae, Cerambycidae and fungal infested trees), were based on permutational ANOVA tests. .

We also checked whether the infestation by bark beetles varies depending on the status and characteristics of the trees. In the GLM model (Poisson error, maximum likelihood, N = 31), the number of infested trees by *Ips* spp. was used as a dependent variable, whereas the log + 1 transformed number of alive trees and the perimeter of the trees served as explanatory variables. The same approach was used to test the relationship between the Cerambycidae , Buprestidae and fungal infestation and the characteristics of the trees. In the models, the number of infested trees by Cerambycidae, by Buprestidae and by fungi were used as dependent variables.

A GLM model (Poisson error, maximum likelihood, N = 31) was built to check whether the beetle or the *Ips* spp. parasitism facilitates the fungal parasitism. In the models, the number of trees affected by fungi was used as the dependent variable and the number of trees affected by beetles was used as an explanatory variable in the first model, whereas the number of trees affected by *Ips* spp. was used as an explanatory variable in the second model.

We were interested in whether a larger number of *F. polyctena* nests provides stronger pest control in the forests by a reduction of the number of infested trees. We used Generalized Linear Mixed Models (GLMMs; binomial error, maximum-likelihood fit, N = 31) to check whether the number of *F. polyctena* nests exert an effect on the number of trees parasitized by each beetle group or fungi. In the models, the ratio of parasitized trees with the *Ips* spp., Cerambycidae, Buprestidae or fungi to the total number of affected trees were used as dependent variables, whereas the number of *F. polyctena* nests as an explanatory variable.

We were also interested in whether the number of *F. polyctena* nests decreases while increasing the dimensions of the nests present within the study plots, and whether larger *F. polyctena* nests reduce more effectively the infestation of the trees by either beetles or fungi. To test the effect of the nest size on the infestation of the trees with the *Ips* spp., Cerambycidae Buprestidae, fungi or by all beetle groups in general, we carried out GLMMs (binomial error, maximum-likelihood fit, N = 31). In the models, the ratio of the parasitized trees (*Ips* spp., Cerambycidae, Buprestidae, fungi or by all beetle groups) to the total number of affected trees were used as dependent variables and the nest size (m^3^) as the explanatory variable. Additionally, we checked whether the number of *F. polyctena* nests influences their size. To test it, we carried out a GLMM (Poisson error, maximum likelihood, N = 31) in which the number of *F. polyctena* nests was used as a dependent variable and the size of the nests (measured in volume, m^3^) as an explanatory variable.

Finally, to test whether the number of the wood ant nests or size have an influence on the number of parasite groups, we used a GLMM (Poisson error, maximum likelihood, N = 31) with the number of parasite groups as a dependent variable and the number and size of the nests as explanatory variables.

The statistical analyses were carried out by using the R statistical software^51^. The RDA (carried out by using the function *rda*) and the PERMANOVA were carried out by using the *adonis2* function in the *vegan* package^52^ and the Hellinger data transformation was performed by using the function *hellinger* from the *labdsv* package^53^. Generalized Linear Mixed Models were built with the *glmer* function from *lme4* package^54^. In all the models, we used the identity of the plots and the study area as nested random factors. All models were tested for model overdispersion by using the *DHARMa* package^55^. If model overdispersion occurred, negative binomial error distribution was used^56^. In these latter cases, the models were built with the *glmer.nb* function. The graphical representation of the results was performed by using the function *ggplot* in the R package *ggplot2*^57^.

## Results

Our study covered a total of 63 ha and was distributed in 12 regions located in a latitudinal gradient of 900 km in beeline crossing three countries. A total of 393 *F. polyctena* nests and 3021 trees were measured and searched for parasite infection (Supplementary Table 1). Considering all the affected trees, 74.9% were parasitized by beetle species. The most common pest species belonged to the bark beetle *Ips* spp., which parasitized 51.3% of the trees.

Our results show that the infestation by *Ips* spp. was not influenced by the increasing latitude, altitude, or the average age of the forests under study. The same was found for the infestation by the *Cerambycidae*, Buprestidae and fungi species (Table 1).

**Table 1:** The effect of latitude, altitude and the average forest age on the number of trees infected by wood boring beetles (Ips spp., Cerambycidae, Buprestidae) and fungi.

Pest groups	Latitude		Altitude		Forest age	
	*z*	*p*	*z*	*p*	*z*	*p*
*Ips* spp.	− 0.10	0.480	0.28	0.777	0.09	0.926
*Cerambycidae*	0.34	0.735	0.24	0.808	− 0.26	0.795
*Buprestidae*	0.38	0.703	0.22	0.825	− 0.30	0.762
Fungi	− 0.34	0.737	− 0.03	0.970	0.14	0.890

The results are based on the GLMM models (negative binomial error distribution, maximum-likelihood fit).

**Figure 1:**
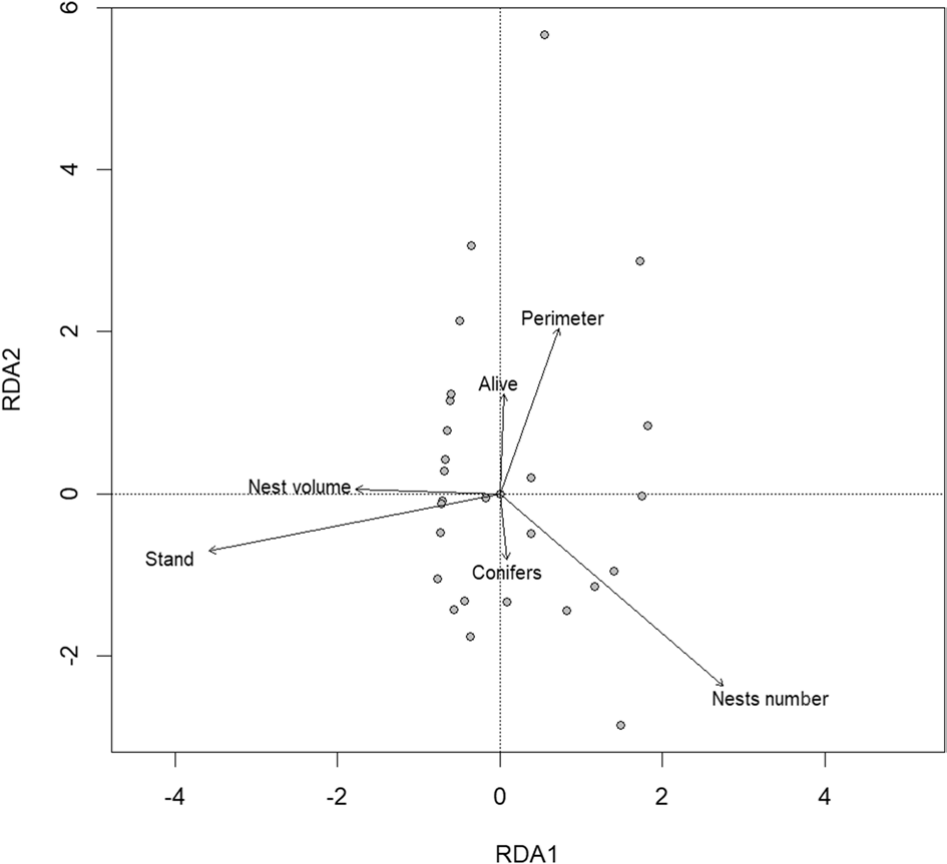
Graphical representation of the RDAs (redundance analyses) on the biotic an abiotic variables.

Overall the results of the Redundance Analysis (RDA) showed that there is a significant relationship (F = 2.29, *p* = 0.001; Fig. 1) between the number of trees affected by the parasites (beetle groups and fungi) and the explanatory variables (Nests Number, Nest Volume, Conifers, Alive, Stand, and Tree Perimeter). The first two RDA axis explained in total 96.44% of the variation of the number of trees affected: the first one explained 86.29%, the second 10.15%. The Nest Number (F = 2.96, *p* = 0.019) and the number of Standing trees (F = 2.46, *p* = 0.051) were significantly associated with the number of trees affected by beetles and fungi, whereas the frequency of Conifer trees, Alive trees, the Perimeter of the trunks or the Nests volume of *F. polyctena* had no significant effect in this respect (Table 2).

**Table 2:** The effect of the number and size of red wood ant nests and tree characteristics (proportion of conifers, alive trees, standing trees and tree perimeter) on the dissimilarities in the number of trees infected by wood boring beetles and fungi.

Characteristics	*R*^2^	*F*	*p*
Nests number	**0.09**	**2.96**	**0.019**
Nests volume	0.01	0.41	0.867
Conifers	0.05	1.61	0.155
Alive trees	0.01	0.28	0.961
Standing trees	**0.07**	**2.46**	**0.051**
Tree perimeter	0.03	0.88	0.478

The results are based on the PERMutational ANOVA.

*Significant results are with bold font.

Moreover, the number of trees infested by *Ips* spp. showed to be negatively related to the proportion of Alive trees but no relationship was found with the Perimeter of the trunk of the infested trees (Table 3). In the trees infested by the *Cerambycidae* and *Buprestidae* groups, we found a positive relationship with the Perimeter of the tree trunks, as well as a negative relationship between the infestation by these groups and the number of Alive trees, which was significant only in the *Buprestidae* infestation. However, no significant results were found in fungal infestations (Table 3). Notwithstanding, our results do not show any significant relationship between the beetle infestation and the fungal infestation in the studied plots (z = 0.29, *p* = 0.775).

**Table 3:** The effect of the tree characteristics (tree perimeter and proportion of alive trees) on the different pest groups (*Ips* and fungi).

Pest groups	Tree perimeter		Alive trees	
	*z*	*p*	*z*	*p*
*Ips* spp.	1.45	0.147	− **5.88**	> **0.001**
*Cerambycidae*	**3.44**	> **0.001**	− 1.71	0.088
*Buprestidae*	**6.26**	> **0.001**	− **3.35**	**0.001**
Fungi	0.31	0.756	0.18	0.858

The results are based on the GLMM models (binomial error, maximum-likelihood fit).

*Significant results are with bold font.

**Figure 2:**
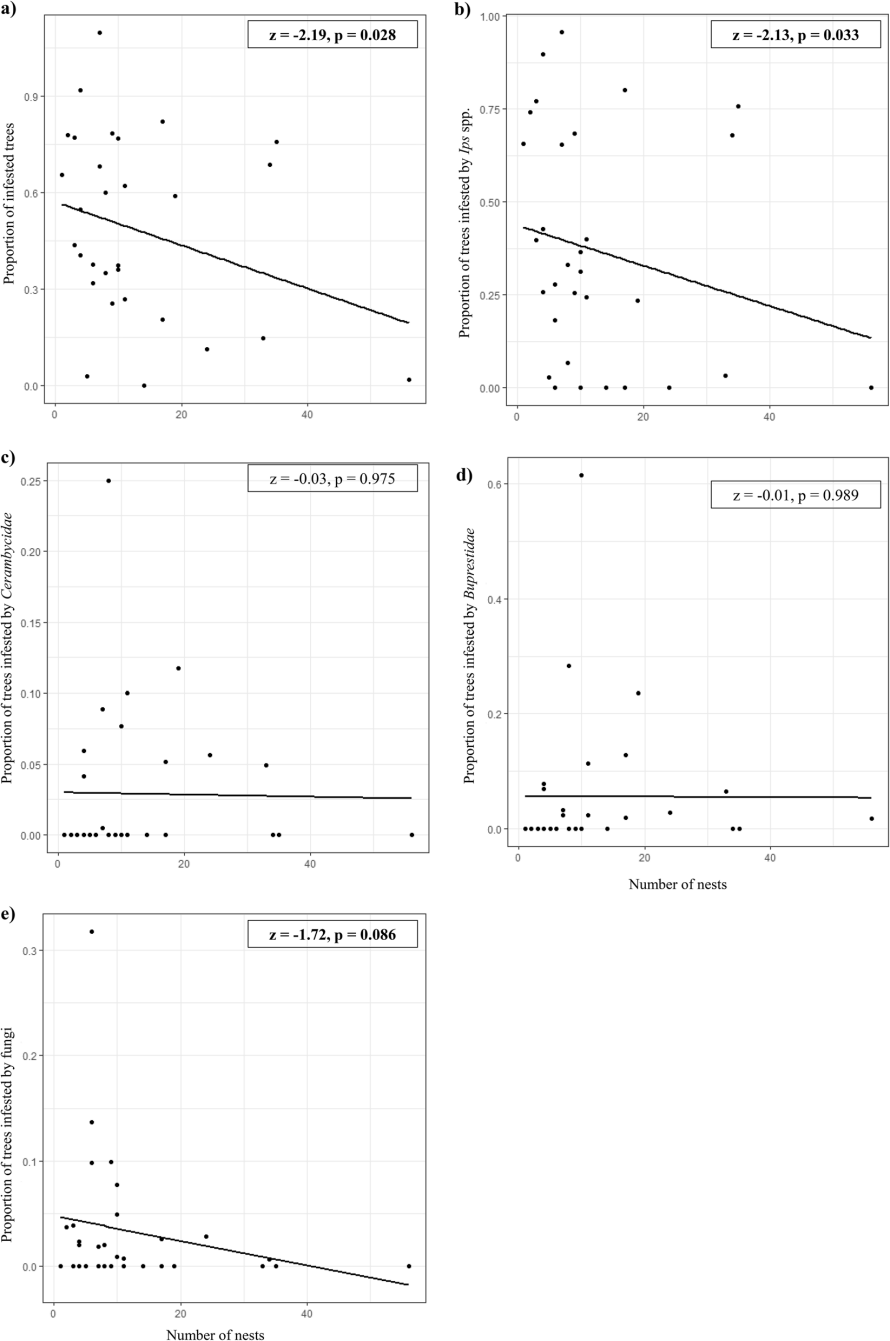
Scatterplot with fitted regression line illustrating the relationship between the number of *F. polyctena* nests and the proportion of infested trees by (**a**) all the studied pest groups, and separately for (**b**) bark beetles (*Ips* spp.), (**c**) boring beetles of the Cerambycidae group, (**d**) boring beetles of the Buprestidae group, and (**e**) fungi. Significant results are with bold font.

**Figure 3:**
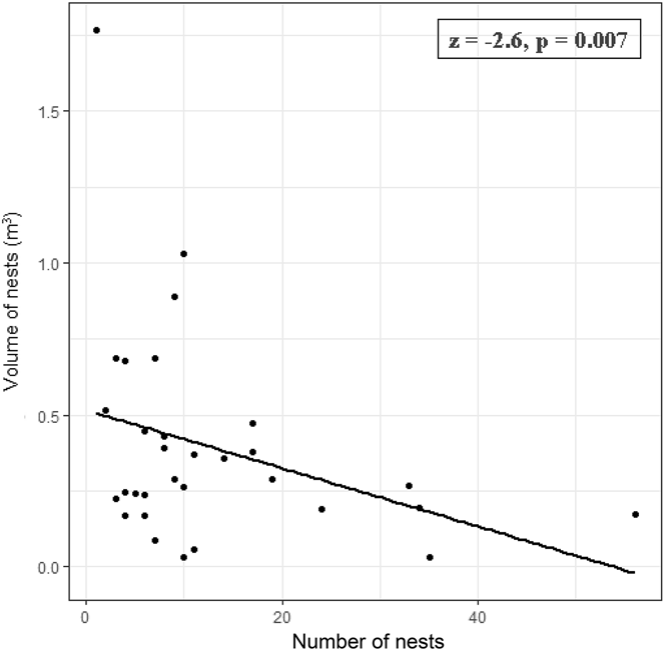
Scatterplot with fitted regression line illustrating the relationship between the number of *F. polyctena* nests and their size.

The increasing number of *F. polyctena* nests present in the study sites was significantly correlated with a reduction in the number of infested trees (z = − 2.19, *p* = 0.028; Fig. 2a). Moreover, the number of infested trees by *Ips* spp. showed to be significantly reduced by the increasing number of *F. polyctena* nests (z = − 2.13, *p* = 0.033, Fig. 2b). However, no significant negative effect of the number of *F. polyctena* nests was found on the *Cerambycidae* (z = − 0.03, *p* = 0.975; Fig. 2c) or Buprestidae (z = − 0.01, *p* = 0.989; Fig. 2d) infestations, on the number of beetle groups infesting the trees (z = 0.05, *p* = 0.961) and on the fungal infestation (z = − 1.72, *p* = 0.086, Fig. 2e). Moreover, the increase in the number of *F. polyctena* nests was correlated with a decrease in their size (z = − 2.60, *p* = 0.007, Fig. 3).

The size of the nests did not have any significant effect on the number of trees affected by *Ips* spp. (z = 1.58, *p* = 0.115), *Cerambycidae* (z = − 0.98, *p* = 0.329) and *Buprestidae* (z = − 1.16, *p* = 0.247) groups separately, nor on the total number of infested trees (z = 0.99, *p* = 0.321). Similarly, an increase in the nest size did not affect the number of trees infested by fungi (z = 0.09, *p* = 0.923). Finally, the number of parasite groups infesting the trees was not affected by the number (z = 0.05, *p* = 0.961) or size (z = − 0.38, *p* = 0.698) of the *F. polyctena* nests.

## Discussion

In our study, we tested the role of *F. polyctena* as pest control for wood-boring beetles and fungi in 12 regions located along a latitudinal and altitudinal gradient from South-Hungary to North-Poland. Our results show that the beetle community was mainly shaped by the number of *F. polyctena* nests, the percentage of alive affected trees and the perimeter of the affected tree trunks present within the studys. The increasing number of *F. polyctena* nests seems to lead to a reduction in the number of trees infected by bark beetles and, despite the fungal infestation was not significantly reduced, it also seems to be affected. However, the number of wood ant nests did not affect the number of trees infected by other species than *Ips *spp. Moreover, neither the nest size, the age of the forests, the altitude or the latitudinal location of the study sites showed to be related to the degree of beetle or fungal infestation.

The infestation by bark beetles is often associated with assemblages of fungi, bacteria, and mites which determine their successful tree colonization and reproduction^28,58^. The death of trees is the result of a double effect: a direct one by the bark beetle boring action and an indirect one by the inoculation with phytopathogenic fungi used to feed their broods^22^. Notwithstanding, the degree of the infestation by these wood-boring beetles can be determined by their local community structure and composition, as found also in other arthropod groups^59,60^. Moreover, the wood-boring beetles, in general, can be regulated also by different habitat characteristics like the health status of the trees, the abundance of host plant species, natural predators or even interspecific competition^61^. From these possible effects, our results indicate that the infestation by the wood-boring species parasitizing the studied forests was shaped to a different extent by the number of red wood ant nests, percentage of alive infested trees and the perimeter of the affected trees.

In our study, though the highest percentage of infestation was carried out by the bark beetles (*Ips* spp.), other wood-boring species belonging to the families *Cerambycidae* (Latreille, 1802) and *Buprestidae* (Leach, 1815) were also present. Due to the higher abundance of *Ips* species, the other wood-boring species would be more successful by avoiding nesting close to the bark beetles to reduce the larval competition for resources^61^, which might result in niche partitioning by the species in their search for an optimal reproduction rate. If we take into account that conifers are the main nesting target for bark beetles^53,62^, and the most abundant trees in our studied forests, this partitioning can be even more accentuated. Furthermore, wood-boring beetles mainly attack dead and weakened trees. Notwithstanding, healthy alive trees and shrubs can be also attacked when the beetle population reaches a high abundance receiving the nomination of “primary invaders”^22,63,64^. For example, *Dendroctonus frontalis*, a *Curculionidae* that produces galleries with an “S” pattern, quickly disrupts the cambium^63^. Moreover, our results show that boring beetles (belonging to Cerambycidae and Buprestidae groups) are benefited by trees with a larger perimeter. Larger trees can offer a more suitable nesting place (higher amount of older tissues) for the boring beetle larvae and adults are more attracted to carry out their oviposition in them. For example, the species of the genera *Agrilus* tend to lay their eggs on the south side of large living trees with thick bark^8^.

The worldwide concept of ants as natural enemies of insect pests^43,44^ is mainly based on the fact that besides their predatory habits, they also disturb pests during their oviposition and feeding^43^. Moreover, they also possess the capability of switching to the most abundant prey and of modifying their foraging behaviour to increase the contact with prey species^65^. In forests, red wood ants can shape the invertebrate community of trees by reducing their species richness through different interactions at multi-trophic levels that also includes the predation on forest pests^21,29^. Additionally, the supercolonies of the red wood ants show characteristics (such as large biomass of supercolonies, long-term stability, colonization of large areas and high predation on herbivores) that make these species even more suitable as biocontrol agents^45,61^. Our results show that the presence of *F. polyctena* nests reduced the number of infected trees by bark beetles. In our scenario, the reduction of this arthropod group might be the result of a combination of a direct effect linked with their predation habits and an indirect effect of the monopolization of resources, the aphid colonies (constituting their stable source of carbohydrates, important in maintaining their large colony size), by the red wood ants^66^.

The complex polydomous system of *F. polyctena* requires a large amount of food that is provided by foraging in mass^29,32^, covering an extensive area of the forest. Moreover, the nuptial flight of bark beetles occurs in spring^67^, coinciding with the presence of the sexual larvae that determines the peak of predation in red wood ants, and the presence of small populations of lachnids, the main food source of red wood ants later on the season^68^. In this scenario, bark beetles can easily become the target prey species of red wood ants, mostly in the spring period. For example, in Białowieża forest has been observed a *F. polyctena* worker with a bark beetle individual in its mandibles (I. Sondej, pers. comm.). Moreover, due to the effects of the climate change (mostly the elevated temperatures), instead of two bark beetle generations in lower latitudes and one in higher latitudes, three and two generations appear, respectively^69^. This can lead to a higher abundance of bark beetles in the spring period, being more available as a food source for red wood ants. Additionally, the vast number of workers moving to the tree canopy to collect the honeydew from the aphids cover almost totality the tree trunks (^34^, authors pers. observ.), making extremely difficult for other arthropods to perch. Moreover, the *F. polyctena* workers tend also to attack insects when they perceive their movement^70^. As a result, the number of available trees for the oviposition of the bark beetle is strongly reduced, so their abundance is as well negatively affected. This can lead to an increase in tree survival and forest health. However, this forest protection effect is local and the protected area would be more accentuated in the springtime and limited to the area covered by the supercolony of *F. polyctena*. The mentioned indirect protection from pests has been already used in agricultural practices, e.g. *F. polyctena* has been used for pest control in apple plantations in Denmark, where its presence led to greater production in the first year^61^.

The presence of red wood ants is strongly linked to habitat features, which is the main reason why the changes in their habitat had strongly affected their populations resulting in their current threatened status^23^. Besides factors like the availability of proper food sources, nesting places, and the material for mound construction^37^, also the temperature and isolation properties are important determinants of the presence of the red wood ant nests both at local and at larger scales^48–50^. Moreover, the combination of the large- and small-scale factors, like the reduction of the insolation on a local scale due to the shading by the tree canopy has been demonstrated to exert an important effect on the *F. polyctena* nests size and distribution^48^. However, the latitudinal and altitudinal location as well as the average age of the forests were not related to the infestation of trees by beetles or fungi. This could be due to anthropogenic climate change which promotes higher generation rates in bark beetle populations^69^ and make possible to these species to become highly abundant in a wide range of habitats.

In conclusion, our study highlights the high effectiveness of red wood ants as pest control agents in mixed coniferous forests. The increasing number of *F. polyctena* nests, regardless of their size, leads to a severe reduction of the number of infested trees, demonstrating the protective role of *F. polyctena* nests mostly against bark beetles. Moreover, it seems that this effect is species-specific and is also influenced by other habitat characteristics. Based on these results we can tell that red wood ants can be valuable tools in the development of forest management plans for the control of pest species, such as *Ips* spp. that are globally a major cause of tree mortality^27,53,71^. Considering that wood ants can be translocated with good success rate (see^72^), their colonies can be reintroduced after restoring the lost habitats where *F. polyctena* was previously present, so they can contribute to their maintenance, or perhaps in not too degraded forest habitats where they might contribute to the recovery of the perturbed forests. Moreover, our study also highlights the importance of the conservation plans for red wood ant species due to their important role in preserving the health of the forests, essential for the forest communities in the light of the threat of global climate change.

## Supplementary Information

Supplementary Information.

## Data Availability

Raw data were generated at Department of Social and Myrmecophilous Insects, at the Museum and Institute of Zoology (PAS). Derived data supporting the findings of this study are available from the corresponding author (GTP) or the senior author (IM) on request.
