# Supplementary material for: Wood ants as biological control of the forest pest beetles Ips spp
Source: Sci Rep. 2021 Sep 9;11:17931. doi: 10.1038/s41598-021-96990-5 (PMC8429415; doi:10.1038/s41598-021-96990-5)
Supplement: Supplementary file 1 — Supplementary Information. [file 41598_2021_96990_MOESM1_ESM.docx]

**Supplementary table 1.** Summarizing table of the number of nests, total number of affected trees, number of standing and alive trees, whereas the number of trees parasitized by the different wood boring (*Ips* spp., *Buprestidae* and *Cerambycidae*) and fungi species in the 12 studied areas.

| **Area** | **Number of nests** | **Number of affected trees** | **Standing trees** | **Alive trees** | ***Ips* spp.** | ***Buprestidae*** | ***Cerambycidae*** | **Fungi** |
| --- | --- | --- | --- | --- | --- | --- | --- | --- |
| Ásotthalom (HU) | 27 | 148 | 95 | 20 | 82 | 5 | 2 | 6 |
| Bialowieza (PL) | 13 | 221 | 103 | 21 | 128 | 0 | 0 | 14 |
| Bükk Mountains (HU) | 52 | 481 | 372 | 15 | 222 | 0 | 0 | 6 |
| Great Fatra Mountains (SK) | 11 | 110 | 73 | 16 | 67 | 0 | 0 | 8 |
| Koszalin (PL) | 56 | 54 | 10 | 1 | 0 | 1 | 0 | 0 |
| Gorce Mountains (PL) | 18 | 562 | 275 | 59 | 332 | 26 | 16 | 1 |
| Kampinos (PL) | 25 | 165 | 133 | 12 | 47 | 0 | 0 | 9 |
| Kiskunság (HU) | 36 | 261 | 59 | 14 | 213 | 23 | 24 | 5 |
| Mátra Mountains (HU) | 52 | 352 | 182 | 101 | 196 | 3 | 0 | 0 |
| Pieniny Mountains (PL) | 13 | 397 | 312 | 91 | 11 | 22 | 14 | 2 |
| Świętokrzyskie Mountains (PL) | 65 | 192 | 92 | 15 | 6 | 23 | 22 | 2 |
| Tatra Mountains (PL) | 25 | 78 | 47 | 1 | 10 | 1 | 0 | 0 |
